# With Crisis Comes Opportunity: Redesigning Performance Departments of Elite Sports Clubs for Life After a Global Pandemic

**DOI:** 10.3389/fpsyg.2020.588959

**Published:** 2021-01-20

**Authors:** Scott McLean, David Rath, Simon Lethlean, Matt Hornsby, James Gallagher, Dean Anderson, Paul M. Salmon

**Affiliations:** ^1^Centre for Human Factors and Sociotechnical Systems, University of the Sunshine Coast, Sippy Downs, QLD, Australia; ^2^St Kilda Football Club, St Kilda, VIC, Australia

**Keywords:** football, complexity, sociotechnical systems, COVID-19, sport, cognitive work analysis, systems analysis

## Abstract

The suspension of major sporting competitions due to the global COVID-19 pandemic had a substantial negative impact on the sporting industry. As such, a successful and sustainable return to sport will require extensive modifications to the current operations of sporting organizations. In this article we argue that methods from the realm of sociotechnical systems (STS) theory are highly suited for this purpose. The aim of the study was to use such methods to develop a model of an Australian Football League (AFL) club’s football department. The intention was to identify potential modifications to the club’s operations to support a return to competition following the COVID-19 crisis. Subject Matter Experts from an AFL club participated in three online workshops to develop Work Domain Analysis and Social Organization and Cooperation Analysis models. The results demonstrated the inherent complexity of an AFL football department via numerous interacting values, functions and processes influencing the goals of the system. Conflicts within the system were captured via the modeling and included pursing goals that may not fully reflect the state of the system, a lack of formal assessment of core values, overlapping functions and objects, and an overemphasis on specialized roles. The current analysis has highlighted potential areas for modification in the football department, and sports performance departments in general.

## Introduction

On the 11th March 2020, the World Health Organization (WHO) declared the COVID-19 outbreak as a global pandemic (World Health Organisation, 2020). In the days and weeks that followed, the global sporting industry was brought to a sudden halt (Evans et al., 2020; Parnell et al., 2020; Toresdahl and Asif, 2020). This began with the National Basketball Association (NBA) in the United States suspending competition after a player tested positive for COVID-19. This was quickly followed by the suspension of all other major sporting competitions, including the world’s biggest sporting event, the Olympic Games, to be held in Tokyo in July 2020 (Sato et al., 2020). To put the situation in context, the scheduling of the Olympic Games has only previously been interrupted due to the second World War. From March 2020 onward, elite sport worldwide entered largely unchartered territory.

The public health crisis associated with the COVID-19 pandemic has been extensively documented by the media, governments, WHO, and via academic commentary and editorials (Heymann and Shindo, 2020; World Health Organisation, 2020). In addition to the public health crisis, is the impending financial crisis brought about by the lock down of entire countries and industries (Goodell, 2020; Zhang et al., 2020). While the overall financial impact of the COVID-19 pandemic is not yet clear (Zhang et al., 2020), short term financial impacts are already being realized within the sporting industry (Evans et al., 2020). As a result of the suspension of play, sporting organizations have been unable to generate revenue from media, memberships, merchandise and ticket sales (Evans et al., 2020). This has led to a requirement to reduce spending, which has contributed to mass unemployment within the sporting industry (Evans et al., 2020). It is anticipated that the public health and economic impacts of the pandemic will be ongoing and will impact sport in both the short and long term (Goodell, 2020; Heymann and Shindo, 2020). As such, the sporting industry will be required to adapt and potentially to re-invent itself upon the resumption of competitive sport (Evans et al., 2020). In the short term, for example, a safe and successful return to play will require significant modifications to current practices such as coaching, training, and injury prevention management. Moreover, severe financial constraints will require sports organizations to revisit the structures and processes currently used to optimize performance. Sporting organizations will need resilience to cope with potential intermittent suspensions in the event of future global pandemics. A successful return to competition will require agility, innovation, and ultimately substantial modifications to current operations to ensure the sustainability of sporting organizations.

In order to understand the inherent complexity of the COVID-19 crisis for sporting organizations, appropriate approaches are necessary. There is a growing body of research applying complexity and systems thinking-based methods to understand and optimize sports systems (Bittencourt et al., 2016; McLean et al., 2017, 2019a; Hulme et al., 2019; Salmon and McLean, 2019; Salmon et al., 2021). Such methods are useful as they can be used to describe sports organizations, their key functions, and the factors that influence performance at the athlete, the team, and at the organizational level. Sociotechnical systems (STS) theory (Clegg, 2000; Read et al., 2018) is one such approach that is used to optimize work systems. It was developed during a program of research undertaken at the Tavistock Institute that focused on the disruptive impacts of new technologies on human work (Trist and Bamforth, 1951; Eason, 2014). The approach encapsulates a focus on both the performance of the work system and the experience and well-being of the people performing the work (Clegg, 2000). Joint optimization, as opposed to optimization of solely the social or technical aspects is required for efficient and healthy system performance (Badham et al., 2006). There is a large body of work demonstrating the positive benefits of adopting STS principles in organizational redesign. A meta-analysis of over 130 STS studies found that almost 90% reported improvements in safety and productivity and over 90% reported improvements in workers’ attitudes and quality of outputs (Pasmore et al., 1982). Although the approach appears highly suited to the design of sports organizations and practices, it is yet to be applied in this context. Despite this, it is our view that STS provides a novel and highly useful approach to support sports organizations in responding to COVID-19.

The aim of this study was therefore to apply methods from an STS framework to analyze the current functioning of an Australian Football League (AFL) club’s football department. The intention was to use the framework to identify potential modifications to the clubs’ operations in the wake of the impacts associated with the COVID-19 crisis.

## Materials and Methods

### Study Design

This qualitative study applied two phases of the Cognitive Work Analysis (CWA) framework (Vicente, 1999), Work Domain Analysis (WDA), and Social Organization and Cooperation Analysis (SOCA) to develop and analyze a complex systems model of an AFL club football department. The WDA and SOCA development were conducted across three subject matter expert (SME) workshops via the Zoom video conferencing software. Five SMEs from the participating AFL club participated in the current study.

#### Cognitive Work Analysis

CWA is a sociotechnical systems analysis and design framework that has been used extensively for understanding the structure and behavior of complex systems (Bisantz and Burns, 2008; Stanton et al., 2017). An important feature of CWA is that it provides a series of analytical methods that focus on identifying the constraints present within a system and the resulting impacts on behavior. This allows analysts to understand what constraints exist, what impact the constraints have on behavior, and how constraints can be modified to improve system performance. The formative nature of the framework allows analysts to explore the possibilities for changing behavior through the removal of existing constraints, the addition of new constraints, or through changing the nature of constraints. These unique features have ensured that CWA has become one of the most popular systems analysis and design methods within the discipline of human factors and ergonomics (HFE), and safety science (Stanton et al., 2017). Recently, CWA has been used across a wide range of domains (Bisantz and Burns, 2008; Stanton et al., 2017), including recently in elite sport for organizational analysis (Hulme et al., 2019), performance analysis (McLean et al., 2017, 2019a), and talent identification and development in soccer (Berber et al., 2020).

The CWA framework comprises five phases, each being used to model behavior from differing perspectives: WDA; control task analysis (ConTA); strategies analysis; SOCA; and worker competencies analysis (WCA). An overview of the two phases used in this project, WDA and SOCA, is provided below.

#### Work Domain Analysis

**FIGURE 1:**
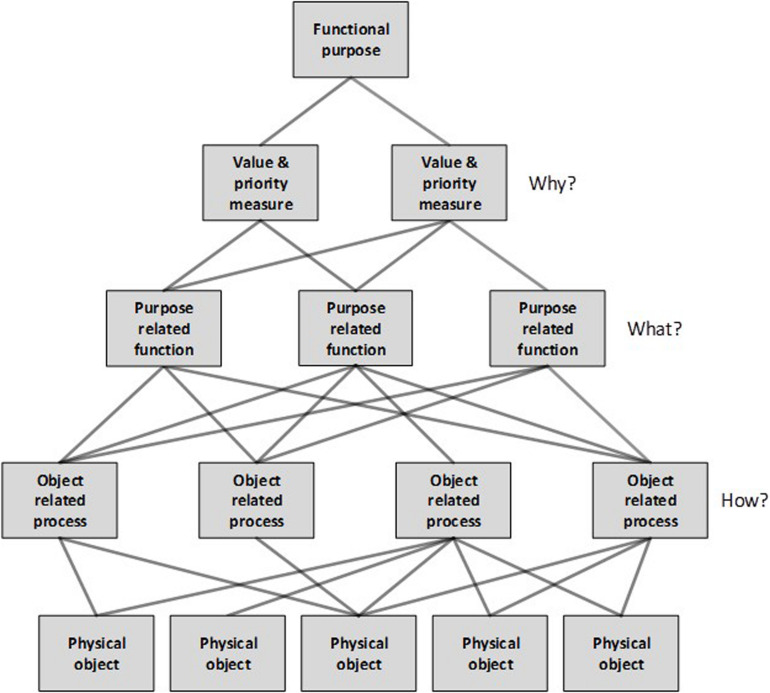
Work domain analysis (WDA) framework showing the levels of abstraction, and the means-end links “how-what-why” triad.

Work domain analysis is used to provide an event and actor independent description of the system under analysis (Figure 1): in this case a current AFL football department “system.” The aim is to describe the purposes of the system and the constraints imposed on the actions of those performing activities within it (Vicente, 1999). This involves using the abstraction hierarchy method to describe the system across five levels of abstraction (Table 1).

**TABLE 1:** Work domain analysis (WDA) descriptions of levels of abstraction.

**Level of abstraction**	**Description**
Functional purpose	The overall purposes of the system and the external constraints imposed on its operation
Values and priority measures	The criteria that organizations use for measuring progress toward the functional purposes
Purpose-related functions	The general functions of the system that are necessary for achieving the functional purposes
Object-related processes	The functional capabilities and limitations of the physical objects within the system that enable the generalized functions
Physical objects	The physical objects within the system that are used to undertake the generalized functions

A key element of the abstraction hierarchy is that it uses means-ends relationships to link nodes across the five levels of abstraction. For example, the object-related process of “Treatment of player injuries” is undertaken to achieve the function of “Injury prevention, management and rehabilitation” and involves the use of the physical object “Medical equipment.” This feature of the abstraction hierarchy enables analysts to understand why functions and processes are undertaken, and what is used to achieve them.

#### Social Organization and Cooperation Analysis

Social organization and cooperation analysis is used to identify how functions and processes are distributed across human and non-human agents within the system. A formative element also enables analysts to determine how functions and processes could be allocated following redesign. By assessing the WDA to identify who/what currently does what, and who/what could do what, SOCA aims to specify an optimum allocation of functions for the system under analysis.

### Procedure

The SMEs had extensive experience in the AFL (16.2 ± 6.1 years), across a range of different roles including players, football director, general manager of football, strength and conditioning, biomechanics, performance analysis, high performance management, AFL governance, coach innovation and education, football strategy and innovation, and playing list management. The SMEs had been employed in these positions at seven different AFL clubs, the AFL, and at the Australian Institute of Sport. In addition, the SMEs had experience in other professional sports including sports science positions in cricket, and tennis. Prior to commencement of the workshops, the SMEs from the participating AFL club’s football department were provided with written information which included an overview of WDA and SOCA as well as a set of preparatory questions for a WDA development workshop. A WDA development workshop was held via Zoom with the SMEs and two researchers with extensive experience in applying WDA (Salmon et al., 2016; McLean et al., 2019a), and the SMEs. The SMEs were asked to respond to a set of WDA prompt questions which were presented in conjunction with relevant keywords and examples [Table 2, adapted from Naikar (2013)]. One researcher used the CWA software tool (Jenkins et al., 2007) to construct an initial draft abstraction hierarchy, using a shared screen function. Following the workshop, the two researchers completed the means-end-links.

**TABLE 2:** Work domain analysis (WDA) development questions and prompts.

**Stage**	**Question**	**Keywords**	**Examples**
1. Functional purposes	Why does the football department exist?	Reasons, goals, objectives, aims, intentions, mission	To win games/grand final Player/team development Implement club strategic plan
2. Values and priority measures	How can we tell whether football department is achieving its purposes?	Criteria, measures, benchmarks	Club reputation Player and team performance Match and Season outcomes Staff and player satisfaction Staff and player retention
3. Purpose-related functions	What functions must be performed by club staff for the football department to achieve its purposes?	Roles, responsibilities, tasks, jobs, occupations, positions, activities, operations	Talent identification and recruitment Performance analysis Coaching and training Load and injury management Manage staff and player health and wellbeing
4. Object-related processes	What processes are physical objects used to achieve within the football department?	Uses, applications, characteristics, limitations, processes	Data collection and analysis Development of physical strength and athletic capacity Communication
5. Physical objects	What physical objects are used within the football department	Tools, equipment, technology, kit, gear, buildings, facilities, infrastructure, staff, people, terrain	Strategic plan Training equipment Gymnasium Finances

A second Zoom workshop was held with the same SMEs to review and refine the draft abstraction hierarchy and undertake the SOCA phase. The SMEs reviewed the WDA components and the means end links. Any modifications were discussed and revised to achieve the final WDA model (Supplementary Material). For the SOCA phase, SMEs were asked to create a list of all actors (employees, consultants, and volunteers) who currently hold a role in the AFL club’s football department. A list of actors was compiled, and the SMEs were asked to identify which of the actors are associated with the Functional Purposes, Values and Priority Measures, Purpose-Related Functions, Object-Related Processes, and Physical Objects specified in the WDA. Actors were identified and associated with the WDA nodes as described in Table 3.

**TABLE 3:** Social organization and cooperation analysis (SOCA) descriptions for the levels of abstraction.

**Level of abstraction**	**Description**
Functional purpose	Actors who contribute to the functional purpose as part of their work in the football department
Values and priority measures	Actors who hold the value and priority as part of their work in the football department
Purpose-related functions	Actors who undertake the function as part of the work in their football department
Object-related processes	Actors who undertake the object-related process as part of their work in the football department
Physical objects	Actors who use the physical objects as part of their work in the football department

The WDA and SOCA were reviewed and refined by the two researchers, following which a third and final workshop was held to complete the SOCA analysis and discuss initial insights from the model. Discussions were documented by the research team and were used to supplement the insights obtained from the WDA-SOCA model.

## Results

### Work Domain Analysis

**FIGURE 2:**
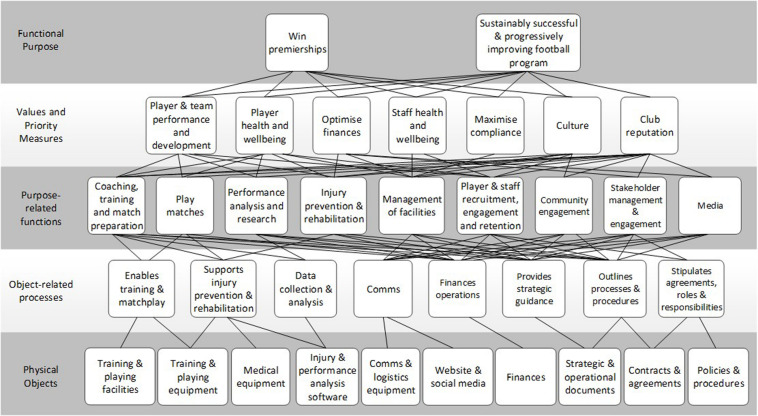
Summary abstraction hierarchy of the AFL club football department. Each level contains summary nodes that encapsulate multi nodes from the overall model. For example, the value and priority measure node of “Player and team performance and development” included “Matches won,” “Percentage differential,” “Continual player improvement,” and “Maximizing player talent.”

The AFL club football department abstraction hierarchy is presented as Supplementary Material. Given the complexity and size of the model, a summary is presented in Figure 2.

According the abstraction hierarchy, the club’s football department has two functional purposes: to “Win premierships,” and to achieve a “Sustainably successful and progressively improving football program.” A total of 25 values and priority measures were identified. These can be broadly grouped into seven categories. The first set includes values relating to player and team performance and development, such as “Matches won,” “Percentage,” “Maximizing player talent,” and “Continual player improvement.” The second set includes values relating to player health and wellbeing, such as “Players physical conditioning,” “Minimizing injuries,” and “Maximizing players health and wellbeing.” The third set includes values relating to club finances, including “Optimizing department spend” and “Optimizing player spend” (salary cap). The fourth set includes values relating to staff health and wellbeing. The fifth set of values relate to compliance such as “Minimizing positive drug tests” (both illicit drugs and performance enhancing drugs) and “Maximizing compliance with AFL rules and regulations.” The sixth set includes values which relate to the development and maintenance of club culture, such as “Player inspiration,” “Player and staff engagement,” and “Embracing and supporting diversity in the playing list.” Finally, the seventh set includes values which contribute to maintenance of the club’s reputation, such as, “Club culture” and “Embrace and support diversity in playing list.”

Forty purpose-related functions were identified. These include functions relating to “Coaching,” “Training and match preparation,” “Playing matches,” “Performance analysis and research,” “Injury prevention and rehabilitation,” “Management of facilities,” “Player and staff recruitment,” “Engagement and retention,” “Community engagement,” “Stakeholder management and engagement,” and “Media.”

At the bottom level of the abstraction hierarchy, thirty-eight physical objects were identified, including “Training and playing facilities” and “Equipment,” “Medical equipment,” “Recovery equipment,” “Performance analysis software,” “Communications and logistics equipment,” “Website and social media,” “Finances,” “Strategic and operational documents,” “Contracts and agreements,” and “Policies and procedures.” According to the abstraction hierarchy, the physical objects support 28 object-related processes including “Training and matches,” “Injury prevention and rehabilitation,” “Data collection and analysis,” “Communications,” “Financial operations,” “Strategic guidance,” “Processes and procedures,” and “Agreements, roles and responsibilities.”

### Social Organization and Cooperation Analysis

A list of the AFL club football department actors considered in the SOCA is presented in Table 4.

**TABLE 4:** AFL club football department actors.

**Code**	**Actors**	**Code**	**Actors**
1	Players	30	Player welfare assistant
2	Leadership group (players)	31	Indigenous welfare
3	Football director	32	COO/GM of football
4	Head coach	33	Head of football
5	Coach	34	Assistant to football department
6	Coach	35	Head of women’s football
7	Coach	36	Head of list management
8	Development coach	37	National recruiting manager
9	Development coach	38	State recruiting manager
10	Coach	39	Opposition analyst/pro scout
11	Leadership consultant	40	Football Program Advisor (list consultant)
12	Leadership and development coach	41	Psychologist
13	Head of Football operations	42	Head trainer
14	Head of Performance	43	Podiatrist
15	Head of sport science	44	Yoga/Pilates
16	S and C coaches	45	Lead football analyst
17	S and C rehabilitation	46	Football analyst
18	Development S and C	47	Football analyst
19	GPS analytics	48	Football analyst
20	Doctor	49	Senior analyst
21	Part time Doc	50	Sleep consultant
22	Part time Doc	51	Legal Counsel and Special Projects
23	Physiotherapist	52	Pastor (part time)
24	Physiotherapist (part time)	53	Massage therapists X 6
25	Physiotherapist (part time)	54	Recruiting administrator
26	Dietician/nutritionist	55	Recruiting scouts x 6
27	Kit man	56	Match day help
28	Facilities manager/staff x 2	57	Analytics interns X 6
29	Player welfare manager	58	Nutrition/S and C interns X 4

*S and C denotes strength and conditioning.*

The results of the SOCA are presented in Tables 5–8. The SOCA results demonstrate how functions and processes are distributed across the actors within the system. Table 5 shows that all actors within the football department are associated with seven Values and Priority Measures, these include Matches won, Percentage, Continual team improvement, Embrace and support diverse playing list, Club culture, Maximize club reputation, Compliance with AFL rules and regulations. Table 6 shows that the coaching staff (actors 4–10), head of performance, sports scientists, the head of football, and the COO/GM of football perform a large number of the Purpose Related Functions relative to other actors. Table 7 shows the Object Related Processes associated with the most actors include Playing games, Enhances physical performance, Protects players, and assessment of player health and well-being. Finally, Table 8 shows the coaches and players utilize the majority of physical objects identified in the abstraction hierarchy relative to other actors. A table is not presented for the Functional Purposes level as all actors were deemed to contribute to both. Within Tables 5–8, the variables associated with each level of abstraction listed in the left-hand side column, with the corresponding columns relating to each the actors from Table 4. Shading is used to denote where actors contribute to each variable. Totals are also presented for the absolute number of actors associated with each value and priority (far right-hand side column) and the absolute number of values and priority measures associated with each actor (bottom row of the table).

**TABLE 5:** SOCA showing the football department actors associated with the values and priority measures identified in the abstraction hierarchy.

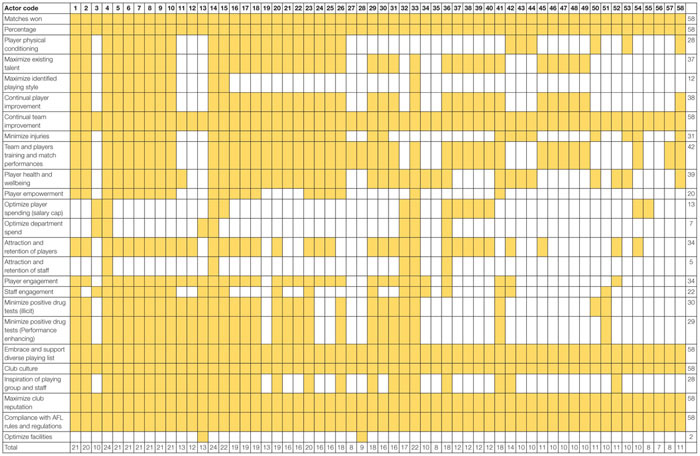

**TABLE 6:** SOCA showing the football department actors associated with the purpose related functions identified in the abstraction hierarchy.

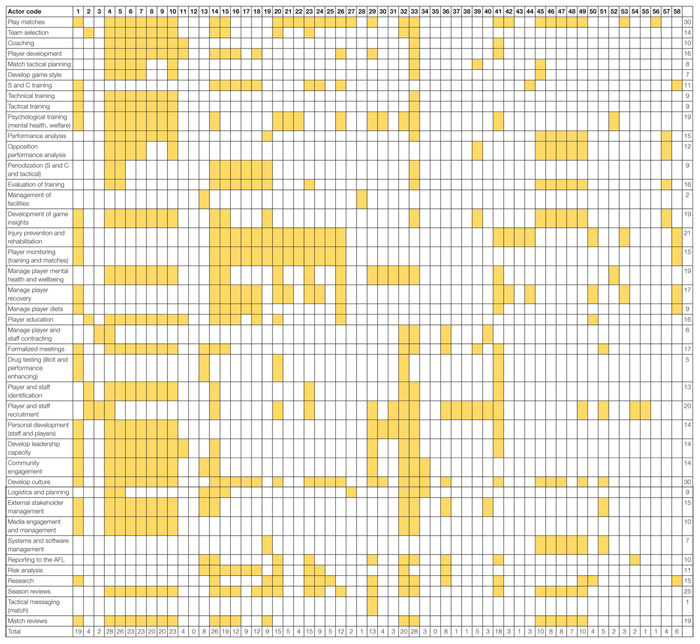

*Within the purpose related functions the playing leadership group (Actor 2) perform the same tasks as the players (Actor 1) in addition to the additional functions shaded.*

**TABLE 7:** SOCA showing the football department actors associated with the object related processes identified in the abstraction hierarchy.

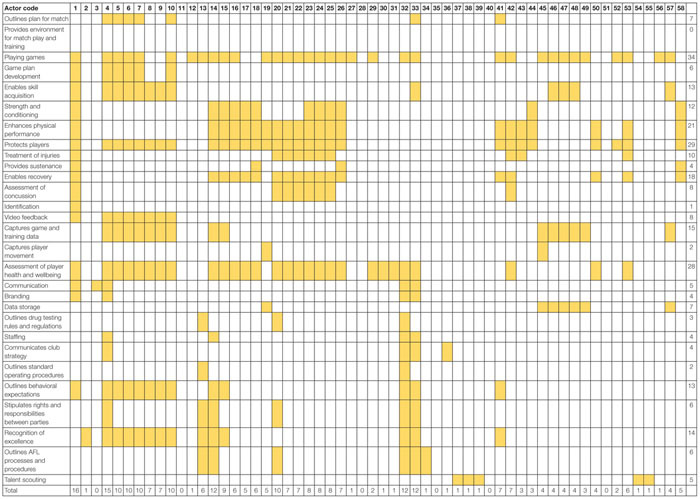

**TABLE 8:** SOCA showing the football department actors associated with the hierarchy physical objects identified in the abstraction hierarchy.

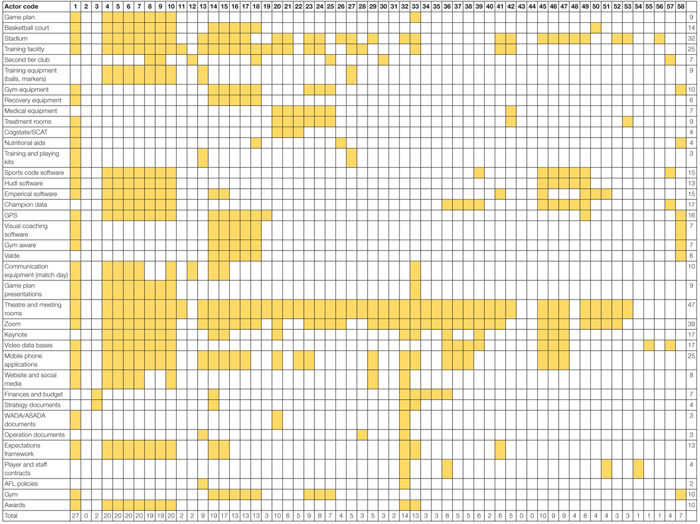

*Within the physical objects the leadership group (Actor 2) uses the same physical objects as the players (Actor 1).*

## Discussion

This study applied two methods from the CWA (Vicente, 1999) framework to provide a detailed analysis of the functional structure of an AFL football department with a view to identifying opportunities for redesign. The analysis produced multiple insights which are relevant for the optimization of sports performance departments in general, and for streamlining current football department operations post COVID-19.

### Complexity of the Football Department

The initial finding of the current study demonstrates the inherent complexity of an AFL football department via the multiple and interacting factors that influence the behavior, and the diverse set of actors who share responsibility for the performance of the system. As such, the department can be conceptualized as a STS in which social actors (e.g., athletes, coaches, facilities staff) interact with one another and with technologies (e.g., equipment, facilities, websites) to achieve common goals (Walker et al., 2008). In addition, the means-end-links in the abstraction hierarchy indicates a high level of connectivity among the system components and behaviors and demonstrate instances where there is either redundancy (i.e., many resources supporting a node above) and where there are potential fragilities (i.e., few resources supporting a node/purpose). These results are consistent with previous research which indicates that sporting organization performance and functioning is an emergent property of the complex interactions between all system components and actors (Jones et al., 2009; Hulme et al., 2019; Salmon and McLean, 2019).

### What Does Systems Modeling Tell Us About the Football Department?

The current analysis identified strengths of the AFL clubs’ department, as well as potential conflicts between systems components. Several strengths of the football department were identified in the WDA including important functions outside of playing football such as community engagement, finances, staff wellbeing, and the development of culture and club reputation (Jones et al., 2009). In line with the aim of the study, the remainder of the discussion will focus on areas for potential improvement and provide recommendations to modify and enhance the club’s current football department in the wake of the impacts associated with COVID-19.

Whilst the two functional purposes are appropriate given the context is elite sport, it seems pertinent to consider the potential adverse impacts of the Functional Purpose of “Win premierships.” As an aspirational Functional Purpose this may be appropriate, however, it is important to note that the AFL club in the current study has endured long periods without achieving this Functional Purpose. Within the study of dynamic system behavior, “seeking the wrong goal” is a common trap made by organizations (Meadows, 2008). If the goal of the system does not reflect the reality of the system, then the system may have problems achieving the intended result (Meadows, 2008). This is known as a stretch goal in organizational behavior research, and although stretch goals can benefit some organizations it appears to be an exception rather than the rule, particularly where achievable and measurable sub-goals are not in place (Sitkin et al., 2011). Stretch goals, compared to more easily achievable goals, increase variance in organizational performance, undermine goal commitment, can adversely impact worker health and wellbeing, and generate lower risk-adjusted performance (Gary et al., 2017). As such, it may be beneficial for the club to set appropriate achievable, attainable and measurable sub-goals which move the organization toward the longer term stretch goal. For example, if at the midpoint of the season the stretch goal of “win premiership” is seemingly out of reach, the overall Purpose can have less of an influence on the behavior of actors within the organization. This is particularly important given that all actors within football department were deemed to contribute to both Functional Purposes. However, if achievable sub-goals become the focus of the organization, progress remains attainable. Without appropriate sub-goals, the realization that the stretch goal will not be achieved mid-season may impact motivation and commitment to the goal. Although stretch goals are attractive to stakeholders, they often produce poor organizational performance (Sitkin et al., 2011; Gary et al., 2017). The use of stretch goals by elite sports organizations is thus only encouraged when appropriate sub-goals are specified and monitored.

The Values and Priority Measures level of the WDA includes the criteria that the football department uses to assess progress toward the Functional Purposes. Many of the measures identified can be used in a straightforward manner to assess progress including “Matches won,” “Percentage differentials,” “Player physical condition,” “Training and match performances.” However, the extent to which data is available to enable the football department to understand whether they are achieving some Values and Priorities is not clear. For example, it is questionable whether valid assessments exist for some of the identified Values and Priority Measures including “Club culture,” “Player empowerment,” and “Maximizing existing talent.” This presents an opportunity for the club to be innovative and develop or adopt new approaches for measuring their progress toward the Functional Purposes. Moreover, the development of valid measures for such aspects of elite sports organization performance represents an important direction for future sports science research.

Many of the measures used within the football department were identified as “lag” indicators in that they are retrospective measures of past performance and outcomes (e.g., “Matches won,” “Percentage,” “Injuries,” “Player improvement”). Within economic and financial modeling and more recently safety science there is an increasing focus on the use of *leading indicators* to help understand and optimize performance. Leading indicators are pro-active measures that allow organizations to predict, for example, safety issues before they arise (Grabowski et al., 2007). An example of a leading indicator in the current WDA includes “Player physical conditioning” which can be a leading indicator for future injuries. It is our view that the development of additional leading indicators for players, the team, the club, and the overall league could have substantial benefit for the football club and elite sporting organizations. This also represents an area for further research, in particular the identification of specific leading indicators and associated measures in different sports contexts.

Despite only a small number of values and priorities relating to the player and football team’s performance itself (e.g., “Win matches,” “Percentage differential,” “Team and player training and match performance”), many of the Purpose Related Functions and Object-Related Processes identified are focused on supporting these values. This is perhaps expected; however, it is worth noting that less support is ostensibly given to other values and priorities such as staff health and wellbeing, culture, and compliance. This suggests that many functions are geared toward winning football matches, with less functions undertaken in support of other values such as developing culture. This highlights a potential conflict between allocation of resources between winning and developing culture. A counterintuitive approach to improving performance may be to redirect resources to developing culture, which has been shown as one of the most prominent factors contributing to successful sporting organizations (Bell-Laroche et al., 2014; Johnson et al., 2014). As such, there may be opportunities to improve the attainment of the lessor supported values as well as those relating to the football team’s performance.

The Purpose-Related Functions identified are largely consistent with those found in previous analyses of elite sporting organizations i.e., “Playing matches,” “Training,” “Performance analysis,” among others (Hulme et al., 2019). An insight derived from this level and the SOCA is the high level of specialist sport science and wellbeing support roles currently used by the football department. This is recognized in previous research with suggestions that professional sports organizations have evolved to encompass an increasingly complex team of specialized experts tasked with a diverse range of responsibilities (Sotiriadou and De Bosscher, 2018; Drust, 2019; Fullagar et al., 2019; Malone et al., 2019). Specifically, the SOCA highlighted the large number of specialist sports science functions and personnel (staff and external consultants) that currently contribute to football department operations. Included are functions such as “Strength and conditioning training,” “Tactical and technical training,” “Psychological training,” “Performance analysis,” “Injury prevention and rehabilitation,” “Player health and wellbeing,” “Management of player diets,” and “Player recovery.” Whilst the intention is to enhance performance, the use of many specialist roles and functions may be viewed differently by coaches and specialists regarding the importance of different activities (Fullagar et al., 2019; Otte et al., 2020). For example, knowledge on tactical and technical expertise is valued by coaches compared to specialist support staff whom often have a vested interest in their own specific area of expertise (Fullagar et al., 2019). This potentially creates the risk of siloed approach whereby the various skill sets are not well integrated (Otte et al., 2020). Given that the coaches ultimately make the decisions regarding training and competition, there is a level of uncertainty around the appropriate utility and value of applied sport science in professional sporting environments (Fullagar et al., 2019). A recent conceptual framework aimed to functionally integrate specialized roles into a multidisciplinary “department of methodology” drawing on expertise of sub-disciplines (e.g., skill acquisition, strength and conditioning) has been proposed (Otte et al., 2020; Rothwell et al., 2020). The purpose is to coordinate activities via shared theory and concepts, communication of ideas, and collaborative design of practice, which may prevent the siloing of sub-disciplines in elite sporting departments (Otte et al., 2020; Rothwell et al., 2020). A further consideration is that the resources required for large teams of specialized support staff and technology (Malone et al., 2019), may not be available post COVID-19. This presents an opportunity to streamline operations by merging roles and enhancing organizational cohesion by moving to a more generalist model whereby general sports science support is provided across these functions by an appropriately skilled but reduced number of personnel (Robertson, 2020).

Another potential negative impact of the large number of specialized support staff employed in professional sport is the conceivable intrusiveness to the players, which is beyond that of most other professions (Drust, 2019). Previous research has shown that a typical English Premier League player has more than 30 individual contacts with performance staff during a normal week (Drust, 2019). Although individual contacts were not measured in this study, the current findings indicate that many of the functions are designed to support player performance, development, and health and wellbeing. Whilst this is important, it also has the potential unwanted consequence of players being overly micromanaged which may diminish autonomy, competence, and subsequently motivation (Ryan and Deci, 2000). For example, the WDA highlights potential conflicts between the micromanagement required for supporting performance, and the functions of “Player empowerment,” “Player education,” and “Develop leadership capacity” in the players. To realize this, the organizational structure should be one that supports the players as the focal point of the club (Kihl et al., 2007), by providing the tools and skills necessary for development. The current findings suggest that, whilst the players are the focal point, many functions appear to be focused on providing direct interventions rather than on empowering players. As indicated in the SOCA, the players do not perform the Functions “Performance analysis,” “Opposition performance analysis,” or “Training evaluation.” Presumably, the players contribute to these functions informally during practice, however, formalizing the involvement of players in these processes may provide autonomy and empowerment.

An insight from the Object-Related Processes level that conflicts with the club’s ambition, is that there are few processes which support the Purpose-Related Functions of “Develop leadership capacity” and “Player education.” Further, the SOCA analysis indicates that the player leadership group currently only undertake four of the 40 functions in the WDA. In the WDA, “Developing leadership capacity” and “Education of players” are linked with several important values and priorities measures above, such as, “Attraction and retention of players,” “Club culture,” and “Player empowerment.” Given the importance of these functions in creating a player led environment, it may be pertinent to explore further ways in which players can be empowered. For example, this may be achieved by enabling player representation in decision making processes and policies that affect the playing group (Thibault and Babiak, 2005; Kihl et al., 2007).

Insights from Physical Objects level of the model included a high degree of overlap in terms of objects and their functionality. For example, “Sports code,” “Hudl,” “Champion Data,” “visual coaching software,” “Strength and conditioning software” are linked to the one process of “Captures game and training data.” While these are all popular tools in sport science and coaching, consideration of how they are being used in terms of the trade-off between cost and resources required for analysis and usability of outputs for coaches is required. This is particularly relevant in light of new financial restrictions introduced following COVID-19. Furthermore, caution is urged with regard to the use of multiple data collection tools and player assessment methods. It is important that the measures do not become a target for the players to pursue at the expense of motivation for training to perform in football matches. This issue has been captured within sports science through Goodhart’s law and the accompanying phrase “when a measure becomes a target, it ceases to be a good measure” (Goodhart, 1984; Strathern, 1997). Assigning importance to metrics can have unintended consequence on behavior which encourages a shift away from the initial intention of improving performance simply to satisfy the metric. For example, the degree of emphasis placed on distance covered, velocity measures (GPS and Gym Aware), the numerous Champion Data metrics (successful possessions, tackles completed, etc.) may have the potential to shift the focus from football performance to its component parts. This form of reductionism is increasingly being recognized as an inappropriate approach for performance analysis and improvement in sport (Glazier, 2010; McLean et al., 2019b; Salmon and McLean, 2019).

The SOCA revealed that there are a high number of actors within the football department. It is out of the scope of this article to determine the appropriate number of actors within the system. However, examination of specific roles is required to determine whether it is feasible to reduce the number of actors whilst still achieving the functions and values and priorities specific in the abstraction hierarchy. A recommendation for other sports organizations is to use methods such as WDA and SOCA to identify opportunities to reorganize their operations in response to COVID-19.

### Implications for Sports Organization Restructure Post COVID-19

The current analysis has highlighted potential areas for modification in the AFL club’s football department, and sports performance departments in general. Whilst it is beyond the scope of the present article to discuss redesigns specific to the club in question, it is possible to prescribe a generic approach that sports organizations can use in response to COVID-19 and other high impact events. First and foremost, it is the authors opinion that redesign activities should be driven by core STS values and principles (Clegg, 2000; Read et al., 2015). Read et al. (2015) outline five core STS design values that appear to be pertinent in the sports context:

1. Humans should be treated as assets rather than as unpredictable, error-prone, and the cause of problems in otherwise well-designed systems.
2. Technology should be used as a tool to assist humans, rather than being seen as an end in its own right (Clegg, 2000; Norros, 2014).
3. Designs should focus on promoting quality of life, rather than creating strict work requirements (e.g., lack of flexibility around working hours and breaks), poor work design (e.g., repetitive tasks, lack of task rotation) and unachievable expectations. Quality work should be challenging and incorporate variety, should include scope for decision-making and choice and facilitate ongoing learning, should incorporate social support and recognition, and should have social relevance to life outside work (Cherns, 1976, 1987).
4. Designs should respect individual differences in the needs and preferences of the various end-users. For example, some players may prefer high levels of autonomy and control, while others may not.
5. Designers should consider all stakeholders, including the impacts of choices they make on various stakeholders. In the present context, these stakeholders include end users, broader club personnel, the community, sponsors, and the AFL.

Based on conducting analyses similar to the one presented in this article, sports organizations could use the prompts presented in Table 9 to identify areas where redesign is required (adapted from Read et al., 2016). Participatory design approaches should then be used in conjunction with the STS values to develop and refine design concepts.

**TABLE 9:** Sociotechnical system design prompts.

**Abstraction hierarchy level**	**Prompt**
Functional purposes	- Are there multiple purposes specified for the system? Do these conflict? Could they potentially conflict? Under what circumstances? - What factors within the system most positively influence the purpose/s? - What factors within the system most negatively influence the purpose/s? - Are any purpose/s of the system not well supported?
Values and priority measures	- Are there conflicting values and priority measures within the system? - Are the value and priority measures currently measured? - Are the value and priority measures currently achieved? - Do different value and priority measures exist in similar systems? - Do the value and priority measures have the potential to encourage functioning that doesn’t support the purpose/s? How?
Purpose-related functions	- Are there any unexpected or unusual functions? - Could any other functions support the purpose/s of the system? - What functions are well-supported by the object-related processes? - What functions are poorly supported by the object-related processes?
Object-related processes	- Are there any unexpected or unusual object-related processes? - Could any other object-related process support each of the functions? - Which object-related processes are well-supported by the physical objects? - Which object-related processes are poorly supported by the physical objects? -
Physical objects	Are there any unexpected or unusual physical objects? - Could any other physical objects support each of the object-related processes? - Which physical objects have the most influence/support the most object-related processes? - Which physical objects have the least influence? - Are any physical objects unreliable in their ability to support the object-related processes? What influence does this have on the system? - How are physical objects related to one another? Do they suffer common mode failures? - Do any objects have the potential to conflict with, or affect the functioning of another object?

## Limitations

The current study contained potential limitations. First, a small number of SMEs were involved in the WDA-SOCA development. However, the SMEs had extensive experience at the current organization and more broadly in the AFL, as well as across several different AFL clubs. Further development and validation of the model and its contents could occur in-house with additional club stakeholders and players. Despite the inclusion of a diverse set of SMEs, potential bias needs to be considered given the financial implications brought about by COVID-19 and the pending restructure to resource allocation within the football department. Further, current players were not included as SMEs in the current study. Given the important role of players within the football department, player input may have provided additional insights specific to the playing group. A second limitation is that the abstraction hierarchy method does not include weightings for the nodes or connections between the nodes across the levels of abstraction. As a result, the relative strength of different nodes and links is not considered and nodes with low incoming or outgoing links could be misinterpreted as being less important or less supported than others (e.g., culture in the present analysis). Whilst this was considered in the present analysis, future research could explore the use of weights to determine the relative importance of values, functions, processes and objects.

## Conclusion

This study applied methods from the CWA framework, WDA and WDA-SOCA, in a first-of-its-kind approach to model an AFL football department. The modeling enabled identification of potential modifications to the clubs’ operations in general, and for streamlining of operations in the wake of COVID-19. The realization of conflicts within the system captured via the modeling will assist the club to redesign operations, and the analysis has important messages for elite sports organizations generally. Firstly, sporting organizations should pursue appropriate goals that reflect the actual state of the system. Secondly, the measures used to assess whether the goals of the system are being achieved need to be specific and measurable in order to obtain valid assessments. Thirdly, shifting to a generalist model that combines specialized roles and objects may increase organizational cohesion, increase system resilience, reduce overlap, and reduce operational costs. This study has extended the applications of systems modeling in sport and provided a practical guide that can be used as template to direct other sporting clubs aiming to redesign their operations. It is hoped that this article emphasizes the important role that sociotechnical systems theory methods can have on sports and sports research.

## Data Availability Statement

The raw data supporting the conclusions of this article will be made available by the authors, without undue reservation.

## Author Contributions

All authors contributed to the conception, model building, analysis, and writing of the manuscript.

## Conflict of Interest

The authors declare that the research was conducted in the absence of any commercial or financial relationships that could be construed as a potential conflict of interest.
