# Supplementary figures and images for: With Crisis Comes Opportunity: Redesigning Performance Departments of Elite Sports Clubs for Life After a Global Pandemic
Source: Front Psychol. 2021 Jan 20;11:588959. doi: 10.3389/fpsyg.2020.588959 (PMC7855175; doi:10.3389/fpsyg.2020.588959)

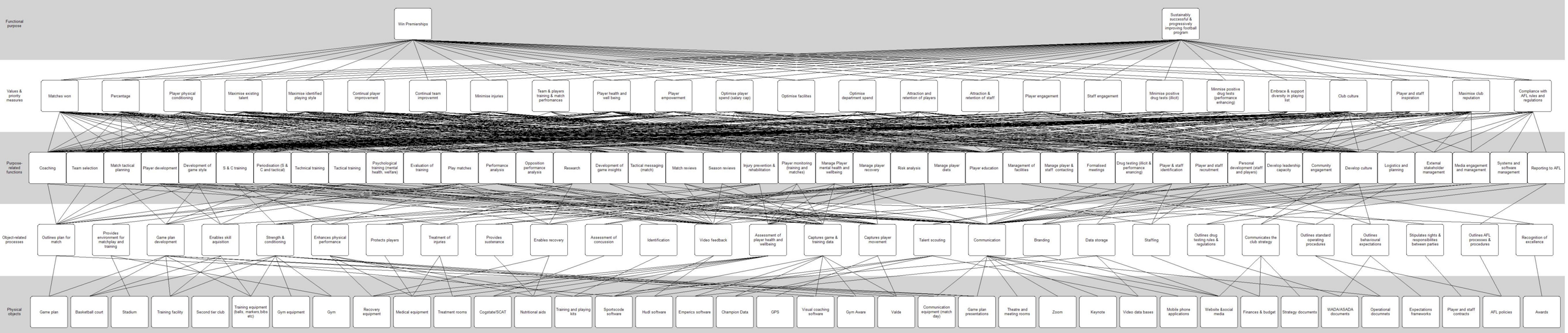

Supplement: Supplementary file 1 [file Data_Sheet_1.PDF]
